# Experimental Analysis of Bisbenzocyclobutene Bonded Capacitive Micromachined Ultrasonic Transducers

**DOI:** 10.3390/s16070959

**Published:** 2016-06-24

**Authors:** Rayyan Manwar, Sazzadur Chowdhury

**Affiliations:** Department of Electrical and Computer Engineering, University of Windsor, 401 Sunset Avenue, Windsor, ON N9B 3P4, Canada; sazzadur@uwindsor.ca

**Keywords:** CMUT, BCB, characterization, efficiency, Polytec™, resonance, velocity

## Abstract

Experimental measurement results of a 1.75 mm × 1.75 mm footprint area Capacitive Micromachined Ultrasonic Transducer (CMUT) planar array fabricated using a bisbenzocyclobutene (BCB)-based adhesive wafer bonding technique has been presented. The array consists of 40 × 40 square diaphragm CMUT cells with a cavity thickness of 900 nm and supported by 10 µm wide dielectric spacers patterned on a thin layer of BCB. A 150 µm wide one µm thick gold strip has been used as the contact pad for gold wire bonding. The measured resonant frequency of 19.3 MHz using a Polytec™ laser Doppler vibrometer (Polytec™ MSA-500) is in excellent agreement with the 3-D FEA simulation result using IntelliSuite™. An Agilent ENA5061B vector network analyzer (VNA) has been used for impedance measurement and the resonance and anti-resonance values from the imaginary impedance curve were used to determine the electromechanical coupling co-efficient. The measured coupling coefficient of 0.294 at 20 V DC bias exhibits 40% higher transduction efficiency as compared to a measured value published elsewhere for a silicon nitride based CMUT. A white light interferometry method was used to measure the diaphragm deflection profiles at different DC bias. The diaphragm center velocity was measured for different sub-resonant frequencies using a Polytec™ laser Doppler vibrometer that confirms vibration of the diaphragm at different excitation frequencies and bias voltages. Transmit and receive operations of CMUT cells were characterized using a pitch-catch method and a −6 dB fractional bandwidth of 23% was extracted from the received signal in frequency domain. From the measurement, it appears that BCB-based CMUTs offer superior transduction efficiency as compared to silicon nitride or silicon dioxide insulator-based CMUTs, and provide a very uniform deflection profile thus making them a suitable candidate to fabricate highly energy efficient CMUTs.

## 1. Introduction

The capacitive micromachined ultrasonic transducer (CMUT) is a type of microelectromechanical systems (MEMS) device that is constructed using processes similar to conventional VLSI technology to generate and receive ultrasound [1]. As opposed to the conventional piezoelectric principle based ultrasonic transducers, the CMUTs rely on electrostatic principles for ultrasound generation and reception. These transducers consume low power, low noise, offer excellent electrical and thermal stability, and a wider fractional bandwidth [1,2,3,4]. Due to these advantages, CMUTs have gained much popularity over the last decade and are considered a potential alternative to commercially available piezoelectric transducers for biomedical imaging, non-destructive testing, high intensity focused ultrasound (HIFU), automotive collision avoidance, and parking assistance applications [5,6,7,8,9]. A typical CMUT geometry is constructed to have a square, circular or hexagonal diaphragm that is supported on a fixed backplate by an insulating layer enclosing a small cavity filled with vacuum or air. The structure functions as a variable capacitor where one of the electrodes (the diaphragm) is free to move or deform against the other (the backplate) to effect a change in capacitance. In the transmit mode, a DC bias voltage superimposed with an AC signal of desired frequency is applied to the CMUT geometry to generate a time varying electrostatic force that causes the diaphragm to vibrate to create ultrasonic vibration in the surrounding medium. When the CMUT is exposed to an incident ultrasound wave, the sound pressure deforms the diaphragm (top electrode) towards the fixed bottom electrode. The resulting change in capacitance is converted into an electrical signal using a suitable microelectronic circuit. Typically, CMUTs are fabricated using a surface micromachining technique or a high temperature fusion bonding process [1,2,3,4]. Silicon nitride or silicon dioxide are commonly used to realize the dielectric spacer in a CMUT structure. However, both silicon nitride and silicon dioxide suffer from high ionic contamination, trapped charges, and wafer bow or cracking during the high temperature annealing process that eventually degrades the transduction efficiency and leads to device failure [10]. The process of silicon oxidation to grow silicon dioxide introduces trapped charges in the Si-SiO_2_ interface due to the interruption of the silicon periodic lattice structure at the Si-SiO_2_ interface [11]. These trapped charges occupy energy states in the silicon energy bandgap. There are also some ionic fixed charges located within approximately 3 nm of the Si-SiO_2_ interface that arise when silicon oxidation stops leaving uncompleted silicon bonds at the interface to result in a sheet of positive charges [11]. The oxidation process also introduces mobile ionic charges, typically alkali ions such as Na, K, and other metallic ions with a surface concentration of 10^12^ to 10^13^/cm^2^ [12]. The combined effects of all these charge contaminations contribute to dielectric polarization at high electric field originating from the bias voltages due to the motion of the mobile ions. For example, the conductivity of the oxide surface was measured to be 10^−12^ S after carrying out the cleaning process [13]. Similar trap charges also exist in silicon nitride thin films arising due to the dangling bonds known as K center defects. Excessive charges trapped in those dangling bonds can alter the C-V characteristics of microfabricated capacitor like geometries such as a CMUT or a MEMS RF switch [14]. In [15], a quasi-two-dimensional simulation and experimental verification of the simulation results of the effects of fixed dielectric charge on the actuation of clamped-clamped beams have shown that a sheet of fixed charge in the dielectric layer results in a voltage offset in the C-V curve of the beam. This offset is directly proportional to magnitude of the fixed charge, while the direction of the shift depends on the polarity of the fixed charge. These results were confirmed by measurements showing a shift in the operational voltages of a microwave switch. As CMUT is functionally a capacitor, like an RF switch, the effective bias voltage will be altered to result in a different bias voltage that is necessary to generate a desired acoustical pressure (transmit) or electrical voltage (receive).

Investigation shows that a low-k dielectric that has been derived from B-staged bisbenzocyclobutene (BCB) and commercialized by Dow Chemical Company [16] under the trade name Cyclotene 3000™, can be used to mitigate the effects of the mentioned charge trapping. In [16,17], it has been reported that BCB or Cyclotene™ has low metal ionic contamination (maximum 10 ppm). Additionally, as BCB can be spin deposited on silicon using AP3000, an adhesion promoter, the possibility of any chemical reaction between silicon and Cyclotene™ that can lead to generation of the mentioned dangling bonds that can hold trap charges will be highly reduced. Furthermore, BCB exhibits a low residual stress of 28 MPa and can be deposited in a low temperature process to minimize wafer bow and cracking [18,19,20]. This also reduces fabrication complexity and cost. The dielectric properties of BCB also exhibit excellent stability over a wide frequency range with a low moisture uptake (0.2%) compared to other conventional dielectrics. It is also worth to mention here that BCB also is an excellent adhesive wafer bonding agent with a fracture strength comparable to silicon [18,19,20]. These observations lead to the conclusion that if BCB could be used as the dielectric spacer between the diaphragm and the backplate of a CMUT, the effects of trapped charges or fixed ionic charges at the interface of the dielectric and silicon can be highly minimized while achieving excellent structural and processing benefits. In [18], the fabrication process of CMUT has been presented where BCB has been used as the dielectric spacer. This paper presents the experimental measurements of the resonant frequency, real and imaginary impedances, coupling coefficient, deflection profile, and pitch-catch mode operation of the device presented in [18]. The measurement results show that the BCB-based CMUT is capable of providing higher transduction efficiency while the close agreement between the measurement and FEA results validates the design process.

The paper has been organized in the following way: Section 2 is a summary of the CMUT design specifications and material properties with some fabrication characterization results using a focused ion beam (FIB) method. Section 3 presents the characterization results that include capacitance and electrical impedance as a function of frequency, coupling coefficient, deflection profile and deflection as a function of frequency, velocity of the CMUT diaphragm, the pitch-catch mode operation results and the frequency response. Finally, the Section 4 presents our concluding remarks.

## 2. Fabrication Characterization of CMUT Array

**Figure 1:**
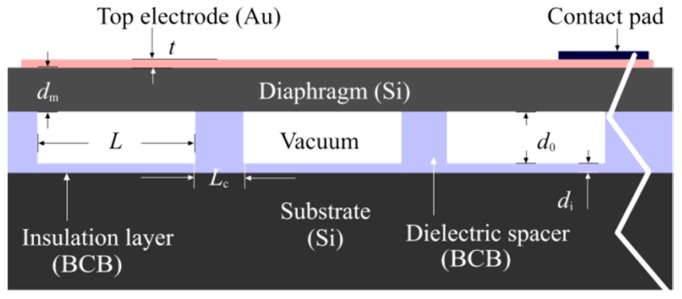
A conceptual cross-section of CMUT array.

A conceptual cross-section of the fabricated CMUT array is shown in Figure 1 and the design specifications are provided in Table 1. Details of the array fabrication process are available in [18]. The physical properties of the materials that were used for fabrication and simulations are provided in Table 2.

**Figure 2:**
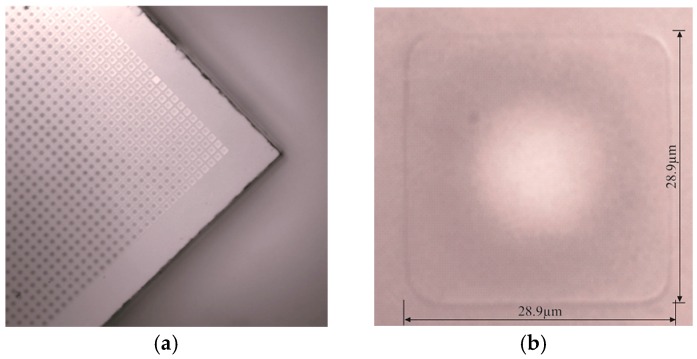
SEM image of: (**a**) A section of the fabricated CMUT array; (**b**) Single diaphragm indentation due to atmospheric pressure.

Figure 2a shows a scanning electron micrograph (SEM) of a section of the fabricated array. Atmospheric pressure indent on the diaphragm of a single CMUT cell is clearly visible in Figure 2b which confirms the near vacuum characteristics of the cavity.

**Figure 3:**
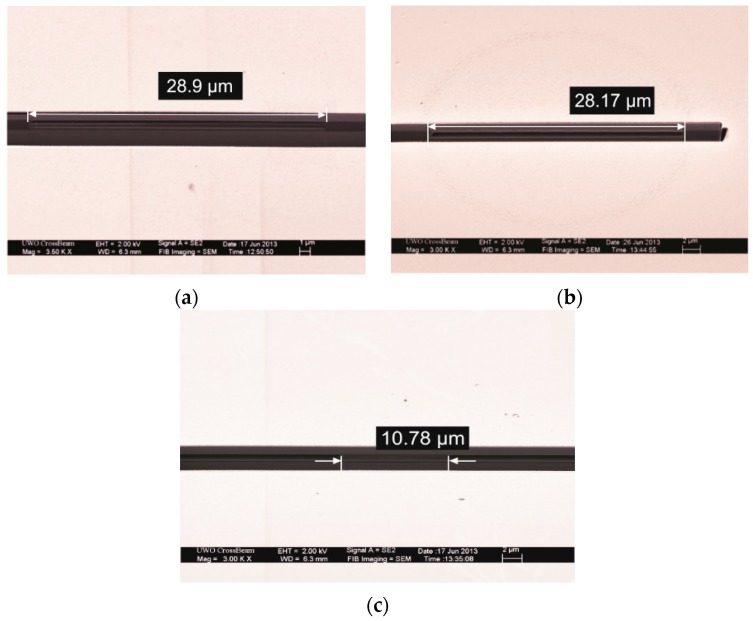
SEM cross-section of the fabricated CMUT array: (**a**) Square geometry; (**b**) Circular geometry; (**c**) Dielectric spacer.

Figure 3a shows an SEM cross-section of one of the square diaphragm CMUTs. The measured 28.29 μm sidelength shows 1.04% deviation from the target specification as listed in Table 1. Figure 3b shows an SEM cross-section of one of the circular diaphragm CMUTs. The CMUT has a measured diameter of 28.17 μm, which is in 0.607% deviation from the design specifications. Figure 3c shows the measured width of the BCB based dielectric spacer. The measured value of 10.78 μm is only 7.8% in deviation from the design specifications. This excellent agreement between the measured values and the target design specifications validates the fabrication process.

## 3. Behavioral Characterization

### 3.1. Capacitance and Imepdance Measurements

**Figure 4:**
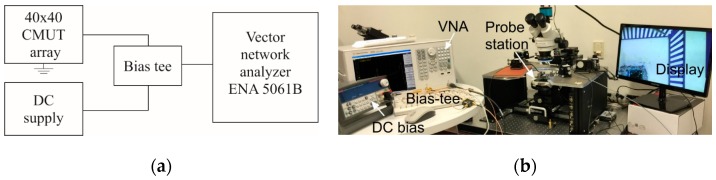
Electrical impedance measurement using VNA set up: (**a**) Schematic; (**b**) Experimental.

**Figure 5:**
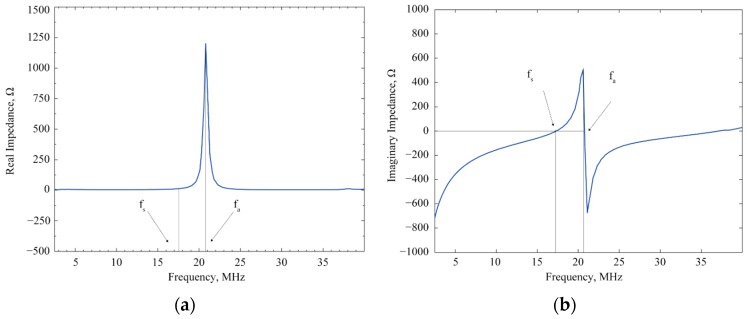
Electrical impedance measurement as a function of frequency: (**a**) Real impedance; (**b**) Imaginary impedance.

The capacitance of one of the CMUT arrays was measured using a Fluke LCR meter (Fluke, Everette, WA, USA). At 10 kHz, the capacitance was 73 pF and at 100 kHz, the measured capacitance was 69.7 pF. Measured parasitic capacitance from the cable and the probe was approximately 50 pF. The real and imaginary electrical impedances were measured using an ENA5061B vector network analyzer (VNA, Agilent, Santa Clara, CA, USA). The measurement setup is shown in Figure 4 and the plots of the real and imaginary impedances are shown in Figure 5. During the measurement, the CMUT was mounted in a PGA fixture from CMC Microsystems (Kingston, ON, Canada). The VNA was calibrated manually with open, short and 50 Ω load at port 1 before taking the measurements. During the measurements, a fixed 20 V DC bias was applied to the CMUT diaphragm using a bias tee (5541A, Picosecond Labs, Beaverton, OR, USA) and the frequency of a small AC signal was swept from 0 to 40 MHz.

The measured S11 parameter was converted into equivalent impedance values using a built-in function of the VNA. The magnitude of the real impedance reaches a maximum of 1.25 kΩ at anti-resonance (f_a_) of 20.6 MHz and a minimum at the series resonance (f_s_) of 17.3 MHz as shown in Figure 5a. Experimental values are in close agreement with the resonant frequency of 16.54 MHz obtained from an FEA simulation carried out using IntelliSuite™ with a percentage deviation of 4.62%. However, it is to be noted here that these values of series and anti-resonance frequencies were obtained at 20 V DC bias that caused a slight downshift of the actual resonance frequencies due to spring softening.

### 3.2. Coupling Coefficient

The electromechanical coupling coefficient $k_{T}^{2}$ that is defined as the ratio of the amount of mechanical energy $E_{m}$ delivered to the load and total energy $E_{t}$ stored in the device can be calculated as follows [21]:

(1) $$k_{T}^{2}=\left(\frac{E_{m}}{E_{t}}\right)$$

where, $E_{t}=E_{m}+E_{e}$. $E_{e}$ represents the electrical energy. Following [21] $k_{T}^{2}$ was extracted from the series resonance and anti-resonance values as measured before using following equation:

(2) $$k_{T}^{2}=1-{\left(\frac{f_{s}}{f_{a}}\right)}^{2}$$

Following Equation (2) and using the series resonance (17.3 MHz) and anti-resonance (20.6 MHz) values extracted from the impedance measurements, the coupling coefficient has been calculated as 0.294. In [21], coupling coefficient of a CMUT was calculated as 0.21 at 40 V DC. Compared to the results presented in [21], the BCB-based CMUT is capable of providing much higher (40%) energy conversion efficiency at about 50% less bias voltage. The static collapse voltage of a CMUT cell with specifications as listed in Table 1 was determined to be 951 V by carrying out a 3D electromechanical finite element analysis (FEA) using IntelliSuite^®^. The FEA result is in excellent agreement with analytical results with 2.8% deviation following a model presented in [22]. The parallel parasitic capacitance of the CMUT was measured as 7.27% of the active capacitance. This value of parasitic capacitance is much smaller compared to the parallel parasitic capacitance (120% of active capacitance) of a silicon nitride based CMUT as presented in [21]. Dielectric charge trapping is a major issue with planar silicon nitride layer that alters the electric field in the gap. Variation of electric field causes higher parallel parasitic capacitance that leads to a reduction in electromechanical coupling efficiency. A silicon membrane based CMUT was presented in [23] where $k_{T}^{2}$ as expressed in Equation (2) was used to calculate the coupling coefficient at 45 V DC bias as 0.072 from the impedance curve as a function of frequency. The comparison shows that the use of BCB as insulation layer and also as a dielectric spacer plays a major role to improve the transduction efficiency.

**Figure 6:**
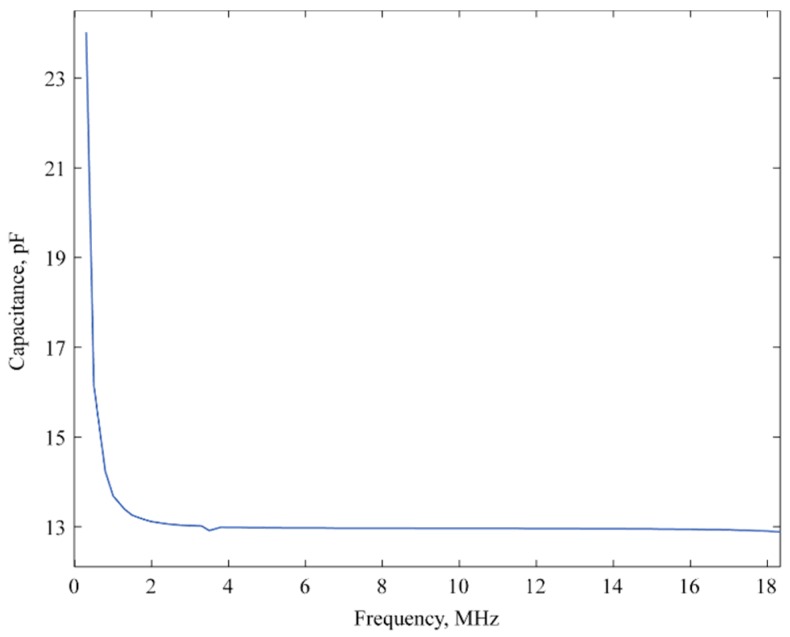
Change in the capacitance due to variation of the diaphragm dielectric property as a function of the frequency.

The capacitance was extracted from the measured imaginary impedance curve and plotted in Figure 6 as a function of frequency. This demonstrates the change in the silicon diaphragm behavior over the specified frequency range of 0 to 18 MHz. Dielectric polarization is affected due to the change in frequency that in turn varies the dielectric property of the CMUT membrane [24]. Consequently, capacitance changes depending on complex dielectric property of the material for different frequencies. The CMUT diaphragm was fabricated using 10 Ω-cm low-resistivity silicon [18]. Figure 6 exhibits the transition of the membrane from conducting at lower frequency to an insulating layer at higher frequency based on the capacitance change [1].

### 3.3. Deflection and Velocity Profile of CMUT Diaphragm

**Figure 7:**
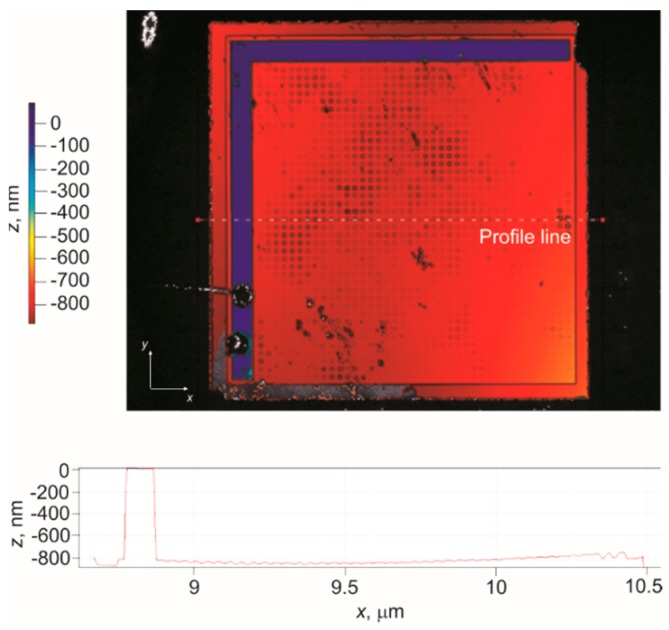
Surface topography of a CMUT array.

Surface topography measurement of the CMUT cells was carried out using a white light interferometer (MSA-500, Polytec™, Irvine, CA, USA). The travel range was calibrated before taking the measurements to achieve maximum displacement. The measurement was done in short coherent mode at 0 V bias voltage. A three dimensional surface topography of a section of the CMUT array is shown in Figure 7. The thickness of the gold contact pad (contact strip) was measured to be 800 nm. A difference of 200 nm from the target specification in Table 2 is possibly due to the non-uniform gold layer deposition using electron beam evaporation during the second metallization step of the fabrication process.

**Figure 8:**
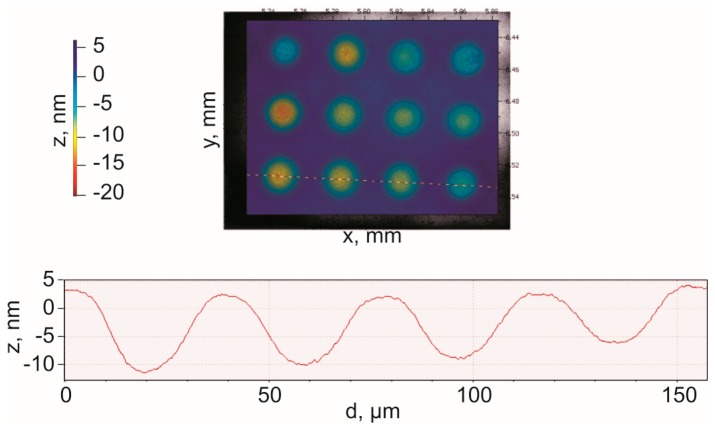
0 V DC bias deflection of CMUT diaphragms measured using a Polytec MSA-500 laser Doppler vibrometer.

Figure 8 shows the deflection profiles of the CMUT diaphragms under 0 V bias condition when measured using a Polytec™ MSA-500. The travel path of the laser beam was precisely set across the center of CMUT cells located in the same row as shown in Figure 8. The zero bias deflection in the range of 10–15 nm as evident from Figure 8 is caused by the atmospheric pressure and the residual stress.

**Figure 9:**
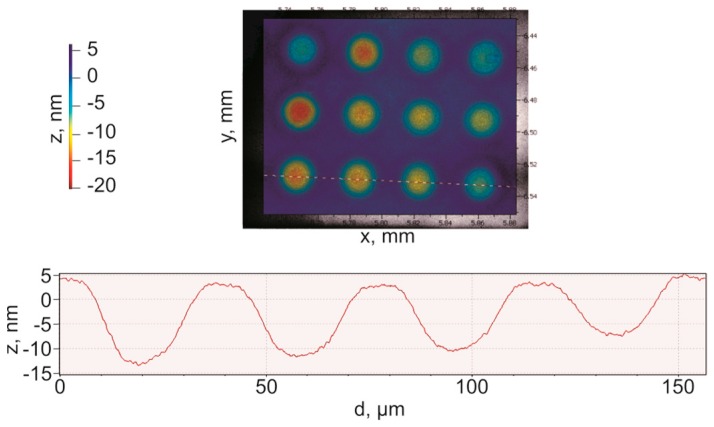
30 V DC bias deflection of CMUT diaphragms measured using a Polytec MSA-500 laser Doppler vibrometer.

The diaphragm deflections due to a fixed DC bias voltage of 30 V measured using the Polytec™ MSA-500 is shown in Figure 9. From Figure 9, an increase of about three nm can be observed which was caused by the electrostatic attraction force generated between top and bottom electrode in the presence of DC bias.

The purchased SOI device layer thickness was 2 µm ± 0.5 µm. To compensate for the wide device layer thickness variation (±0.5 μm) of the purchased SOI wafers, the device layer thickness of each SOI wafer was measured at many points and the average was used to adjust the etching time in an STS RIE equipment to obtain a uniform thickness of 800 nm as best as possible over the entire wafer [18]. However, dry etching down to 800 nm across the 100 mm wafer surface that has a variation of ±0.5 µm was quite challenging. SEM measurements show that there were some nanometer scale variations of the final device layer thickness that ultimately translated into slightly different stiffness for the diaphragms of different CMUT cells. Though the membrane thickness and gap height closely matches with the design target, these variations in stiffness among the membranes eventually affected the deflection profile under same bias and lead to different deflection profile.

**Figure 10:**
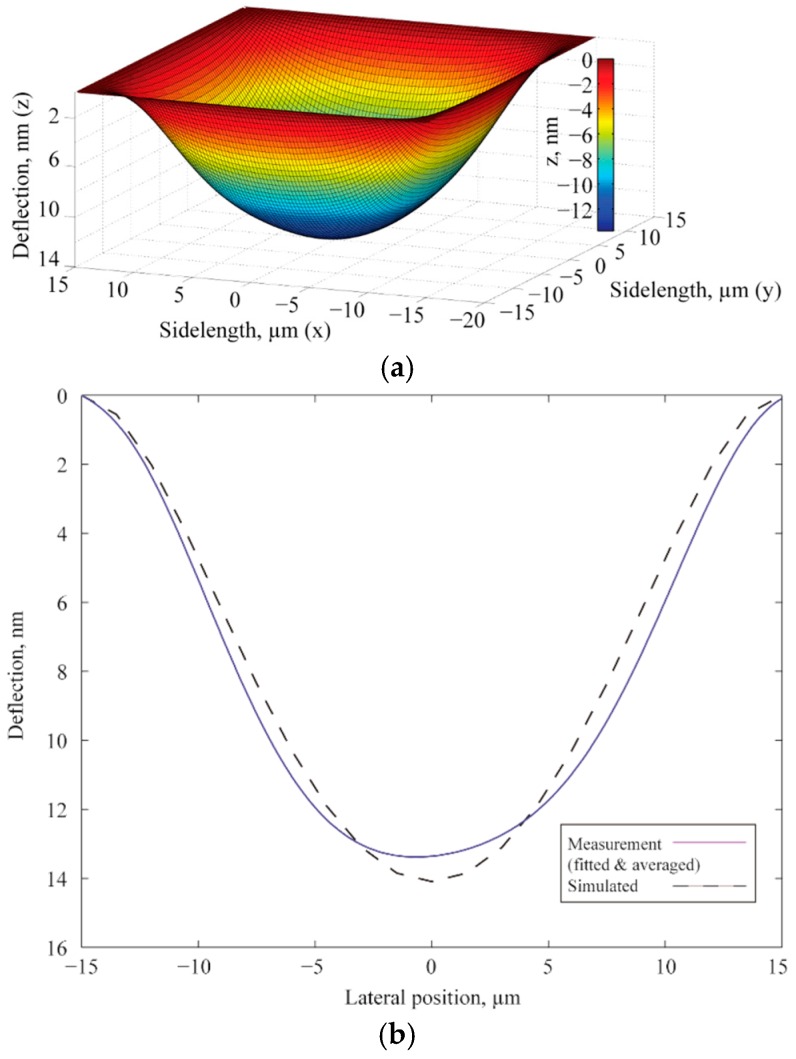
At 30 V DC biasing: (**a**) Simulated deflection of a CMUT cell diaphragm; (**b**) Fitted and averaged deflection profile measurement using MSA 500 compared to the simulated result.

CMUT cell deflection has been compared with the FEA model designed in IntelliSuite™ with a load of 30 V DC bias and shown in Figure 10. The FEA model was implemented by selecting fixed edges as the boundary condition of the square diaphragm shown in Figure 10a. Deflection curves of four randomly chosen CMUT cells have been smoothed using second order polynomial curve fitting model and averaged in MATLAB for an overall fit. A maximum deflection of 13.2 nm is shown in Figure 10b at the center of the diaphragm with a deviation of 4% from the simulated result due to the difference in thickness among the cell diaphragms.

**Figure 11:**
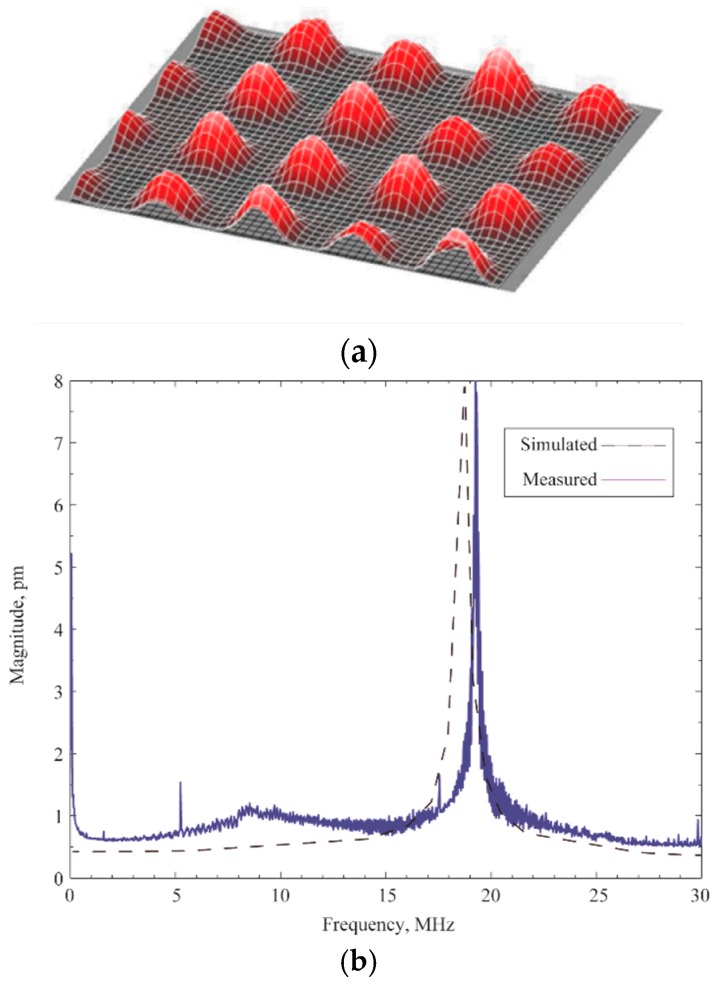
(**a**) Polytec™ MSA-500 screen capture of the deflection vs. frequency measurement of a section of the CMUT array at 10 V chirp signal; (**b**) Deflection vs. frequency plot shows the resonance occurred at 19.3 MHz, nearly twice the applied signal frequency of 9.5 MHz.

The displacement vs. frequency plot of the CMUT array while operating in air has also been carried out using the Polytec™ MSA-500 where a 10 V chirp signal with a frequency sweep from zero to 15 MHz has been applied at 0 V DC bias. The diaphragm is supposed to deflect the maximum at the second harmonic of the applied signal frequency in the absence of a DC bias due to non-linear electrostatic force equation in Reference [1]. A 3-D snapshot of a section of the array with vibrating diaphragms was captured from Polytec™ MSA-500 laser Doppler vibrometer is shown in Figure 11a. In Figure 11b, the maximum deflection of the diaphragm center has been measured as 7.8 picometers at 19.3 MHz when the frequency bandwidth was 30 MHz.

**Figure 12:**
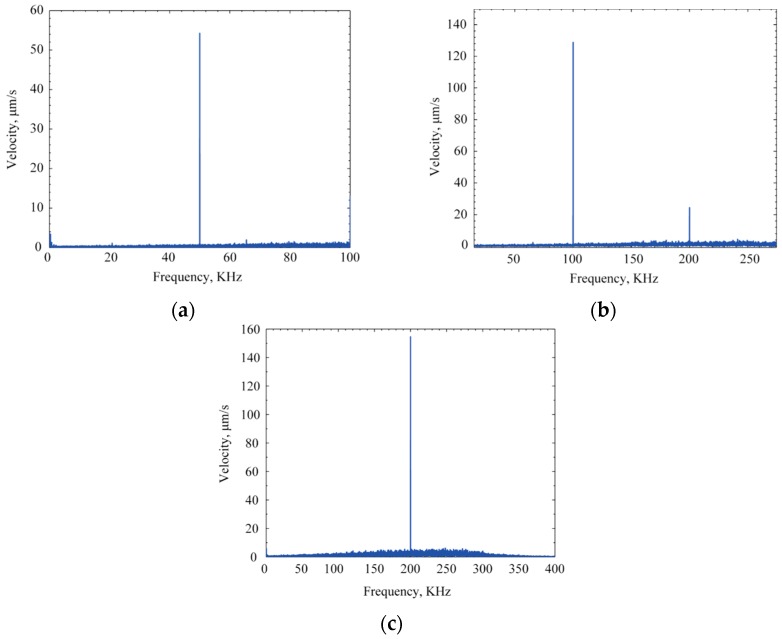
Velocity measurement of CMUT diaphragm at lower frequency than resonance: (**a**) 50 kHz; (**b**) 100 kHz; (**c**) 200 kHz.

Velocity of the center of the CMUT diaphragm was measured with a 20 µm diameter laser from a Polytec™ laser Doppler vibrometer. The frequency range of the actuation signal was 50 to 200 kHz with a bias voltage of 25 V and peak-to-peak sinusoidal actuation signal amplitude of 20 V. A velocity of 54.6 µm/s can be observed in Figure 12a when the signal frequency was at 50 kHz. Figure 12b shows the measured velocity of the center of the diaphragm as 120 µm/s at 100 kHz with a harmonic at twice the actuating frequency. 158.2 µm/s velocity of the diaphragms was measured at 200 kHz signal frequency and shown in Figure 12c. Figure 12 clearly establishes CMUT operation at different frequencies and bias voltages.

Two key observations can be made from this experimental characterization. One is the validation of the transmit operation of the CMUT structure even at a much lower frequency than the resonance. The other is the increase in the magnitude of the velocity while the frequency was increased.

### 3.4. Pitch-Catch Mode

**Figure 13:**
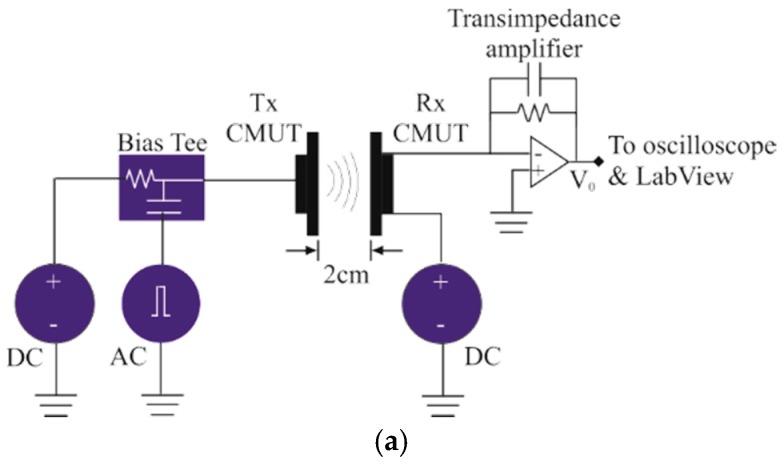

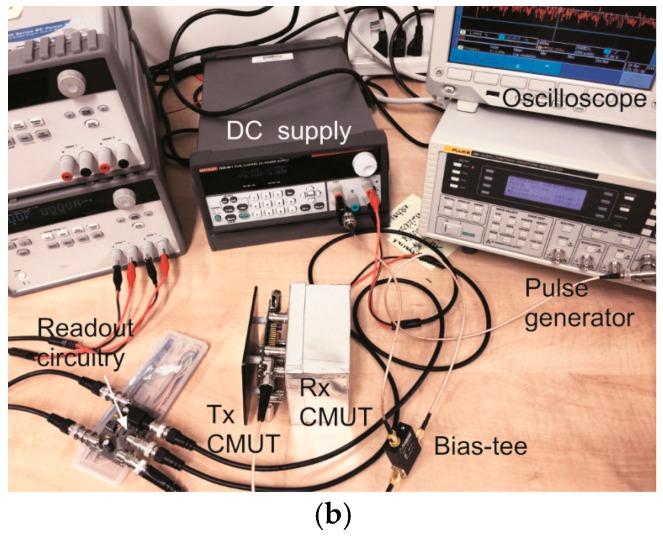
Pitch-catch mode measurement set up: (**a**) Schematic; (**b**) Experimental.

The CMUTs were also characterized using a pitch-catch mode experiment to experimentally verify the receive and transmit operations using one of the CMUT array as the transmitter and the other as a receiver. The distance between the CMUT arrays was 2 cm. The test setup is shown in Figure 13.

**Figure 14:**
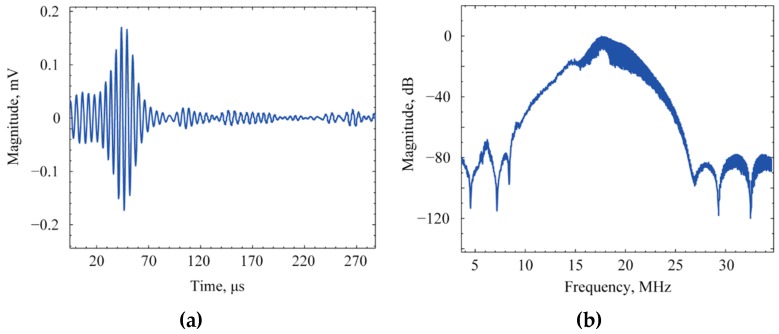
Pitch-catch measurement results in: (**a**) Time domain; (**b**) Frequency domain after averaging and filtering.

An arbitrary waveform generator (Fluke 294) was used to excite the transmit CMUT array using two 29.4 ns pulses with a peak amplitude of 5 V. Both the CMUT array was biased at 30 V using dual channel power supply (2220-30-1, Keithley, Beaverton, OR, USA). The received signal was amplified in trans-impedance configuration using a low noise amplifier (LT1122). A digital oscilloscope (DPO 3032, Tektronix, Beaverton, OR, USA) was used to digitize and average the received signal 128 times. After transferring to a host computer through an USB cable the averaged signal was filtered and a FFT with Hanning window was carried out using NI LabView Signal Express Tektronix Edition 2.5.1. The received signal is shown in Figure 14a after averaging and the normalized frequency spectra is shown in Figure 14b that shows a −6 dB fractional bandwidth of 23.5% and a Q-factor of 4.25. The ringing at the end of the received signal is due to the shielding of the circuit board and small frequency mismatch of transmit and receive transducers.

## 4. Discussion and Conclusions

The experimental analysis of a newly developed dry etched BCB (Cyclotene™)-based CMUT array has been presented. BCB has been used as the inter-electrode dielectric and also as a low temperature adhesive wafer bonding agent to realize a simple CMOS compatible wafer bonding. The low ionic contamination of BCB resulted in a 40% higher transduction efficiency for the BCB-based CMUT as compared to published results using silicon nitride- or silicon dioxide-based insulators. The Polytec™ laser Doppler vibrometer analysis showed a very consistent velocity profile of the diaphragm center at different frequencies and bias voltages.

Pitch catch mode measurements verified the transmit and receive operations of the fabricated CMUTs. Though the measurements were done in air, the CMUTs can also be used for immersion applications, as there is no perforation in the diaphragms. Use of the adhesive wafer bonding would also reduce the fabrication cost. The functionality of the CMUT structure realized from BCB based adhesive wafer bonding technique can be improved further in terms of wider frequency bandwidth and lower parasitic capacitance effect. The frequency response can be improved by using BCB as the membrane material. The dielectric properties of BCB has much more stability for wide frequency range. Also, a thinner insulation layer will contribute to lower the parasitic capacitance. The parasitic capacitance can also be reduced by introducing integrated readout circuitry that can minimize the signal to noise ratio with proper impedance matching. Instead of using the DIP package, flex pcb can be used for mounting the array that will reduce the parasitic capacitance as well as the impedance mismatch. Lower impedance mismatch will also contribute to improve the frequency response.

## Figures and Tables

**Table 1:** Design specifications of CMUT structure.

Parameters	Values
No. of cells	1600 (40 × 40)
Cell diameter/sidelength, *L*, *(2a)/*μm	28
Dielectric spacer width, *L_c_*/μm	10
Diaphragm thickness, *d_m_*/nm	800
Cavity height, *d_0_*/ nm	900
Insulating layer thickness, *d_i_*/nm	200
Top electrode thickness, *t*/nm	100
Contact strip (pad) width, *w_c_*/μm	150
Contac strip (pad) thickness, *t_c_*/μm	1

**Table 2:** Material properties.

Parameters	Cyclotene™ (BCB)	Gold (Au)	Low Resistivity Silicon 100 (Si)
Young’s modulus, *E*/GPa	2.9	70	165
Poisson’s ratio, *ν*	0.34	0.44	0.26
Density, *ρ*/kgm^−3^	1050	19,300	2329
Residual stress, *σ*/MPa	28	106	55
Relative permittivity, *ε*	2.6	6.9	11.8
